# Intracranial hypertension after rosacea treatment with isotretinoin

**DOI:** 10.1007/s10072-023-07039-6

**Published:** 2023-08-30

**Authors:** Johannes Reifenrath, Christian Rupprecht, Vincent Gmeiner, Bernhard Haslinger

**Affiliations:** Department of Neurology, Klinikum Rechts Der Isar, Technical University of Munich, Ismaninger Str. 22, 81675 Munich, Germany

## Background

Idiopathic intracranial hypertension (IH) also known as pseudotumor cerebri is a neurological condition characterized by increased intracranial pressure in the absence of liquor circulation disorders [1]. It usually occurs in obese female patients aged between 20 and 50 years and manifests through holocephalic headache, retroorbital pain, and visual impairments [1]. Although most cases of IH are idiopathic, secondary forms can be a consequence of medication side effects, e.g., of tetracyclines [2], isotretinoin [3], or the combination of both. Here we present a rare case of recurring IH under systemic isotretinoin monotherapy and subsequently under systemic doxycycline monotherapy, possibly additionally triggered by a preceding COVID-19 infection. Optical coherence tomography (OCT) imaging and MRI are correlated to the diagnosis and therapy.

Isotretinoin is a vitamin A derivative applied for the treatment of severe nodular acne and rosacea [4]. It impairs comedogenesis by reducing hyperkeratosis and sebum production and creates thus a less favorable microenvironment for *Proprionibacterium acnes* [5].

Up to 90% of patients under isotretinoin treatment report dry skin and other dermatologic conditions [6]. Other common side effects include unspecific headache, conjunctivitis, mucosal infection, back and muscle pain, and laboratory changes of liver enzymes, blood cells, cholesterol, and triglycerides [6, 7]. In rare cases, isotretinoin seems to affect also the peripheral and central nervous systems. Known side effects of doxycycline are gastrointestinal symptoms, photosensitivity, onycholysis, and IH [8]. Especially the combination of isotretinoin and tetracyclines may result in IH [9] and is therefore highlighted by a warning in the package insert. However, the question whether also a monotherapy with systemic vitamin A derivatives such as isotretinoin can cause IH is less clear. Although a survey suggests that IH incidence is increased upon treatment with tetracyclines [2], only few case reports suggest the occurrence of IH after systemic monotherapy with vitamin A or isotretinoin [10].

## Case presentation

**Fig. 1:**
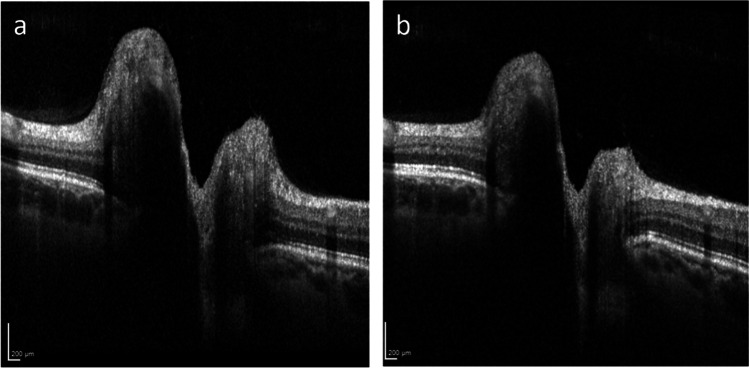
Optical coherence tomography of the left eye reveals decreased papilledema after treatment with single liquor drainage and discontinuation of isotretinoin. **a** Initial swelling on the day of hospital admission. **b** Follow-up 1 month after treatment

A 52-year-old female with a BMI of 22.5 kg/m^2^ was referred to our hospital due to bilateral papilledema and a global retro-orbitally pronounced headache. Initially, the patient consulted an ophthalmologist because of a conjunctivitis of the left eye. Fundoscopy showed bilateral papilledema, and a subsequent OCT confirmed a protrusion of the optical discs (Fig. 1a). The patient reported no acute visual impairment. Two weeks before the patient had developed a persisting global, retro-orbitally pronounced headache during a brief COVID-19 infection. At the time of admission to the hospital, the patient was tested negative for COVID.

The patient had suffered from severe rosacea conglobata for at least 3 years. Previous medication, including four doxycycline prescriptions over the past 2 years last applied 1 year ago, had shown only marginal effects. Two months prior to hospital admission, a rosacea treatment with oral isotretinoin 20 mg daily was initiated.

**Fig. 2:**
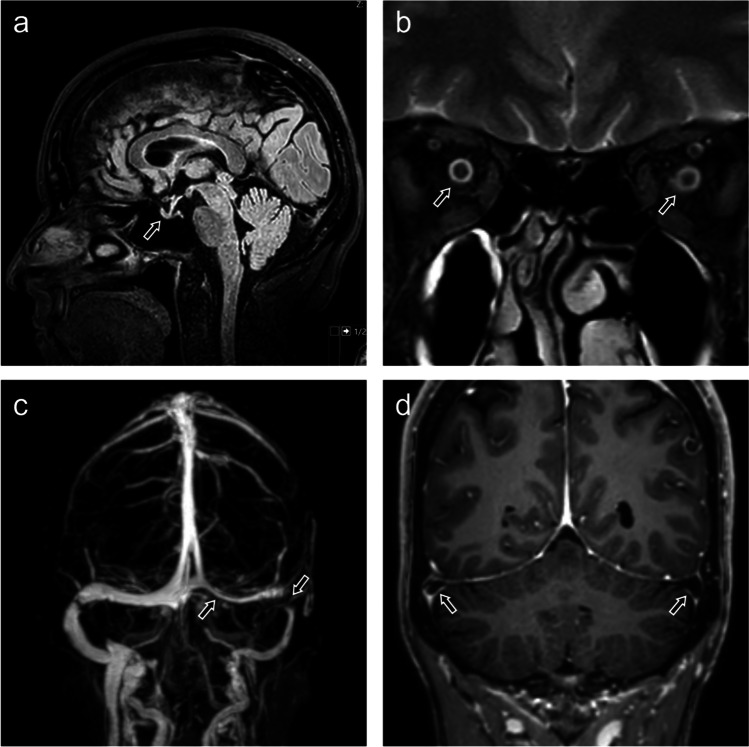
Four classical signs for IH: cranial MRI images of the patient at the time of admission. **a** Flattened sella (arrow). **b** Bilaterally swelled optic sheaths (arrows). **c** Venous angiography with stenosis of the right transverse sinus (arrow). **d** Prominent arachnoid pouchings in both transverse sinuses (arrow)

Cranial MRI revealed characteristic radiographic features of IH, such as thickened optic sheaths, a partially flattened sella, stenosis of the left transverse sinus, and prominent arachnoid outpouchings (Fig. 2). Concomitant lumbar puncture revealed an increased intracerebral pressure of 28-cm water column.

A single therapeutic liquor drainage of 30 ml led to marked improvement of the headache within 4 h, compatible with the diagnosis of IH [1]. Two days later, the patient was discharged with a prescription of 250 mg acetazolamide twice daily. Isotretinoin treatment was terminated.

One month later, the patient presented to the hospital again with a bilateral, pressing headache of moderate intensity. This headache started 4 days after the first lumbar puncture. In addition, the patient complained about tinnitus. She did not mention any exacerbation of the pain when standing up but confirmed suspension of her regular work out due to a headache. A control OCT showed persisting but decreased papilledema. Due to the latter, we performed a control lumbar puncture that showed a normal intracranial pressure of 18 cm cerebrospinal fluid. No liquor was drained.

After another 4 months, the patient was referred to the hospital for a third time because a follow-up fundoscopy had shown increasing papilledema again. Patient history revealed that 6 weeks earlier, a therapy with oral doxycycline 40 mg per day had been administered against the recurring rosacea. An OCT confirmed the swelling of the papillae (data not shown). In a lumbar puncture, we measured 26-cm water column. No liquor was drained. Doxycycline was suspended, and the patient discharged with 250 mg acetazolamide twice daily.

Six weeks after the last episode, the patient reported a significant improvement in the headache.

## Discussion and conclusions

Headache is considered as a common side effect of isotretinoin. According to the package insert, 1–10% of patients complain of headache during isotretinoin intake [11]. Although usually this is considered as an uncomplicated side effect, it may constitute a serious neurological adverse event in rare cases. Here, we report on a patient with initially unspecific headache after intake of isotretinoin, which we later attributed to an underlying condition of IH.

The combination of tetracyclines and vitamin A derivates is known as a contraindication because of the risk of developing IH. In the case of headache and suspected IH, diagnostics with MRI and fundoscopy/OCT are recommended. To our knowledge, this is the first report temporally relating OCT-data to IH under systemic isotretinoin monotherapy.

Epidemiological studies and first case reports indicate that monotherapy with vitamin A or its derivates may also induce IH; however, the picture is less clear. An increased incidence of IH after all-trans-retinoic acids application has been reported during the treatment of acute promyelocytic leukemia [12] or after excessive vitamin A consumption [13]. Moreover, a survey linked 179 out of 1.950 cases of pseudotumor cerebri to isotretinoin use and even observed a positive rechallenge in 6 cases [3]. However, it did not discriminate between a monotherapy and combination with tetracyclines. 

Here we document the appearance of IH under initial monotherapy with isotretinoin. The interval between the last doxycycline and first isotretinoin prescription in our patient was 11 months. Since the half-life of doxycycline is around 24 h [14], any interference of the tetracycline and isotretinoin can be excluded.

Based on the existing literature, there could be an association between idiopathic IH and a COVID-19 infection [15, 16]. While the precise pathophysiology link is not yet understood, preliminary findings suggest the possibility of a cerebrospinal fluid outflow obstruction as a consequence of systemic inflammation [17]. Consistent with the presumed pathomechanism, the initial symptoms of COVID-19 IH manifested in close temporal proximity to the onset of COVID-19 symptoms. Since the headache onset in our patient was also temporally related to preceding COVID-19 infection, isotretinoin therapy and systemic inflammation triggered by COVID-19 might have synergistically contributed to the development of IH. This hypothesis is further corroborated by the fact that the patient had not developed IH in response to a previous doxycycline administration, the last applied 1 year before the COVID-19 infection. As the headache symptoms showed a prolonged course far beyond the healed COVID-19 infection and a discontinuation of acne therapy resulted in reduction of the papilledema, we still see the administration of isotretinoin as decisive.

As such, for patients with persistent headache under treatment with isotretinoin, secondary causes should be considered, especially if the patients present with atypical symptoms or the duration of the headache exceeds more than 2 weeks. In patients with suspicion of primary IH, a medication review should be performed. This should be done in particular in the case of atypical age or habitus. In case of abnormalities, liquor circulation disturbances, e.g., sinus thrombosis, should be excluded by cranial MRI and the diagnosis of IH confirmed by lumbar puncture. According to the AWMF Guidance Manual, a change of dermatological therapy to, e.g., azithromycin or ivermectin can be considered.

In a follow-up 4 weeks later, the patient reported headache of different character with a clear onset 4 days after the initial lumbar puncture and accompanying tinnitus. A second lumbar puncture was performed to monitor the decrease in the intracranial pressure. With the intracranial pressure in the physiological range, we saw no connection to the underlying disease and rated the symptoms as a possible post-dural punctual headache [18]. The reduction in intracranial pressure upon termination of isotretinoin corroborates the medication as the putative cause (diagnosis ex juvantibus).

Four months later, the patient presented again with retroorbital pain and slightly increased intracranial pressure after prescription of doxycycline. We believe this episode of IH to be a known side effect of the tetracycline [19] and suspended the causing medication.

Although our case report suggests an isotretinoin induced IH when the patient initially presented, some limitations should be considered. First, the temporal correlation between isotretinoin treatment and IH is no proof for a causal relationship. The clinical and ophthalmological improvement of our patient following cessation of isotretinoin treatment may also be due to lumbar puncture and temporal treatment with acetazolamide. Moreover, the subsequent recurrence of the IH under doxycycline treatment suggests a high idiosyncratic susceptibility to IH in this patient in general, possibly further triggered by a COVID-19 infection.

## Data Availability

Data is available upon reasonable request.

## Abbreviations

IH: Intracranial hypertension
OCT: Optical coherence tomography
